# Self-Diffusion in Two-Dimensional Colloidal Systems: A Computer Simulation Study

**DOI:** 10.3390/e27111091

**Published:** 2025-10-22

**Authors:** Piotr Polanowski, Andrzej Sikorski

**Affiliations:** 1 Department of Molecular Physics, Faculty of Chemistry, Lodz University of Technology, Zeromskiego 116, 90-543 Lodz, Poland; 2 Faculty of Chemistry, University of Warsaw, Pasteura 1, 02-093 Warsaw, Poland

**Keywords:** anomalous diffusion, colloids, dynamic lattice liquid, lattice models, Monte Carlo method

## Abstract

The dynamics of dense colloidal systems are not fully understood. In the study of these types of systems, computer simulations based on the so-called hard sphere model play a significant role. In the presented work, we consider a system of hard spheres of the same size but different mobilities (molecules with high mobility correspond to solvent molecules, while molecules with reduced mobility are colloid particles) at varying concentrations. For this purpose, a two-dimensional lattice and an thermal model of such systems was designed. In order to determine the properties of such systems, a Monte Carlo computer simulation was used, employing the Dynamic Lattice Liquid (DLL) algorithm. Our main aim was to determine how the dynamic behavior of the system in the short time affects the long-time behavior. For this purpose, we investigated the cross-ratios of the diffusion coefficients in the short and long time of the considered system elements. It was found that the reduction in the solvent mobility with increasing concentration of colloidal particles in a short time leads to a very similar reduction in the mobility of the colloid particles in a long time, but we do not observe such behavior in the case of the solvent, i.e., there is a decrease in the value of the solvent diffusion coefficient in the long time with the change in the concentration of colloid particles, but it is difficult to connect it in a simple way with the decrease in the diffusion coefficient in the short time.

## 1. Introduction

Colloidal systems consist of mesoscopic particles with sizes ranging from 10^1^ to 10^3^ nm suspended in a solvent. These particles are much larger than the solvent molecules, but small enough that their Brownian motion is not disturbed by other mechanisms of motion, such as gravitational sedimentation or convection. The Brownian motion of colloidal particles is driven by random interactions between solvent molecules, direct interactions and hydrodynamic forces, mainly transmitted indirectly through the solvent between colloidal particles, also occur. The basic experimental tools used to study the statics and dynamics of colloidal suspensions are static and dynamic light scattering (SLS and DLS) and optical microscopy [1,2,3,4,5,6,7,8,9,10,11,12,13,14].

The dynamics of colloidal systems are far from being completely understood, mainly because they extend over a wide range of time and length scales due to the difference in size, shape, and mass of the colloids and the solvent molecules, giving rise to complex and long-ranged hydrodynamic interactions. Simulating the dynamics of even monodisperse colloidal systems is not a simple task [11,12,13,14,15,16,17,18,19,20,21,22,23,24,25,26,27,28,29,30,31,32,33]. This is because we generally deal with very high-density systems; moreover, direct interactions between colloid agents and solvent-mediated hydrodynamic interactions should be taken under consideration [7,9,10,14,17,26,27,28,29]. The last problem is related to the close correlation of elements involved in the movement. Many valuable results regarding the dynamics of such systems have been obtained within the framework of the hard sphere model [18,34]. The natural simulation method that implements this model is the event-driven molecular dynamic method [19,35,36]. Unfortunately, due to the very high densities (taking into account the presence of the colloid and solvent), simulations of large systems of this type are very time-consuming. Attempts were made to overcome these known difficulties using simplified methods based on Brownian dynamics [20], but the use of these methods usually hinders the direct observation of hydrodynamic interactions. Hydrodynamic interactions in such systems are frequently mentioned in the literature [24,25,26,27,28,37,38,39,40]. Their impact on diffusion in the short and long time is debated. In reality, it is difficult to obtain a clear picture of what these interactions actually look like. A clear picture of interactions in this kind of system can be provided by the Dynamic Lattice Liquid (DLL) algorithm proposed by us, based on the concept of cooperative movements, which is the result of random attempts to move individual elements and the interaction of excluded volumes and the result of which, in the form of a loop, cooperative movements can be observed directly in the simulation [41].

In this work, we continued computer simulations of two-dimensional monodisperse colloidal systems. In our previous work, we studied systems in which the colloid particles differed in size from the solvent and were immobilized [42]. The system in the current work corresponds to hard disk systems in which the size of the solvent and dispersion medium particles are the same, but the mobility of the particles dispersed in the solvent varies significantly in relation to the mobility of the solvent (which may result, for example, from differences in the masses of the solvent and dispersion medium molecules). In our case interactions between colloid agents and solvent, mediated hydrodynamic interactions (HI) are explicitly taken under consideration and manifested as loops of cooperative movement which can be directly monitored during simulation. In contrast to other simulation works dealing with such issues in which HI have been ignored, our model based on DLL treatment can give a fuller picture of the dynamics of real colloidal systems in a wide range of concentrations of the dispersed substance. By observation of mean-squared displacement of simulated molecules, we obtained coefficients of short- and long-time diffusion in the case of solvent molecules and dispersed agents and the relationships that exist between them. Moreover, the analysis based on the study of cooperative motion loops allows for the establishment of relationships between diffusion coefficients and hydrodynamic interactions.

## 2. Models and Methods

There are many methods described in the literature for simulating colloidal systems, which differ from each other in the degree of accuracy with which hydrodynamic interactions between colloidal particles are taken into account. As a rule, the use of a particular method is the result of a compromise between the expected results and the cost of calculations. In simulations of colloidal systems, a frequently used method is Langevin dynamics, which is a modification of Brownian dynamics. This modification consists of adding to Newton’s equation of each particle a linear Stokes friction term and its corresponding random term to simulate the effect of the surrounding elements of colloid. This method can only be used with very low colloid concentrations because it ignores inter-particle HI. To some extent, these limitations can be overcome by using so-called Stokesian dynamics, but this modification is very time-consuming. In some situations, involving molecules with more complex geometries and an explicit solvent, Dissipative Particle Dynamics is used [43]. The above techniques are more general and powerful but also require very long observation times. It should be emphasized that in all the above methods, direct observation of hydrodynamic interactions, if possible, at all, seems to be very difficult. This is due to the fact that the time of significant displacement (by the size of a molecule), even in simple liquids, is much longer than the time of a single interaction. Only a combination of multiple interactions leads to significant displacement, which we can attribute to hydrodynamic interactions. In other words, the time after which we observe the result of hydrodynamic interactions is short, but long in relation to the time of a single interaction.

Taking the above into account, a method is needed that allows us to bypass the formation time, which is important from the point of view of hydrodynamic interactions. The method by which this task can be accomplished is the DLL algorithm, based on the concept of cooperative movements. The use of the cooperative movement concept on which the DLL algorithm is based allows us to reduce the configuration space and simplify the interactions, and to significantly speed up the calculations. Moreover, the information we obtain by analyzing the statistics of cooperative movements gives quite a clear picture of the effects of hydrodynamic interactions in the system [44]. The DLL algorithm has been described in numerous works on the dynamics of simple liquids [45], disordered environments [35,46], polymer solutions [47,48,49], reaction fronts [50], and polymerization [51,52]. Here, we will only summarize the most important information about this algorithm.

**Figure 1:**
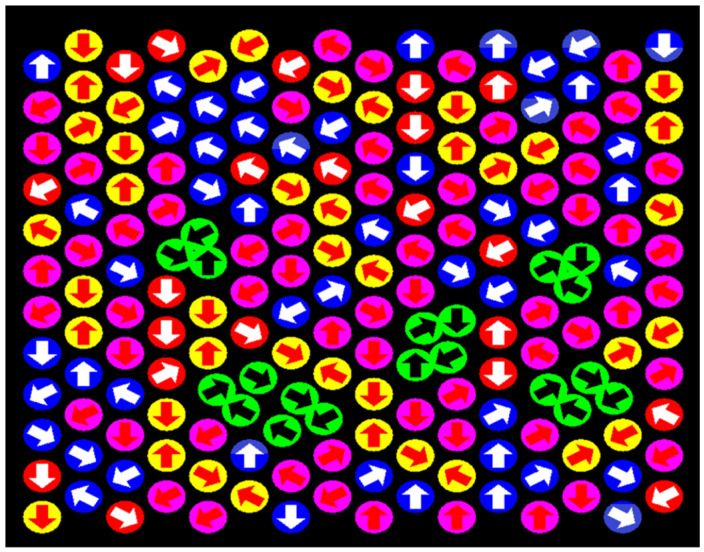
Illustration of one step simulation generated by DLL algorithm for simple liquid.

The configuration space of this algorithm is typically a regular triangular lattice in two dimensions or a face-centered cubic lattice in three dimensions (this choice is associated with a high coordination number and high packing density). In the presented work, we consider the two-dimensional case of a simple liquid. The basic Monte Carlo step (the time step) of the DLL algorithm presented in Figure 1 consists of three consecutive operations: (1) A random field of vectors is generated, corresponding to attempts to move to neighboring lattice nodes (arrows drawn on disks) (2) Impossible movements due to the interaction of excluded volumes are eliminated (disks marked in blue, yellow, red, and pink) or inability of participation in cooperative motion loops (disks marked in red) (3). Shift along the detected cooperative motion loops (the sum of vectors in these loops is equal to 0, disks marked in green). It should be emphasized that although the algorithm is executed on sequential machines, its nature is strictly parallel. Moreover, it operates in a fully populated state, meaning that all lattice nodes are populated by elements corresponding to fluid particles, which leads to a close correlation in the motion between individual elements [35]. It is easy to see that cooperative loops which are a natural consequence of correlated motion in a dense system vary in length, which indicates varying ranges of hydrodynamic interactions, i.e., the longer the loop, the greater the range of hydrodynamic interactions (a more detailed analysis of the loop length distribution and the probability of their formation depending on specific conditions will be conducted later in this paper). Observing this type of behavior within another simulation model seems to be very difficult (if it is possible at all) because it would require building very complex correlation functions depending on the position and time of the elements participating in the simulation.

The system under consideration consists of two kinds of elements, which have differing mobilities [50]. The mechanism of the introduction of differing mobility of elements in the case of the DLL model is described in detail in [42]. In the present work, objects called B are particles for which the probability of participating in a cooperative loop is decreased when compared to mobile molecules of liquid, called A. Below we refer to particles A as particles of solvent and to particles B as particles whose mobility is much lower than the mobility of the solvent molecules. The only potential introduced into the model is the excluded volume of objects realized as prohibiting the double occupancy of lattice sites. The model system used in simulations consisted of 256 × 256 elements with periodic boundary conditions employed along the x and y axes. Results were averaged after at least ten runs. The concentration of colloid particles (B) with reduced mobility was defined as a fraction of lattice sites occupied by particles with the reduced mobility, i.e., *ϕ_B_* = *n*/256^2^, where *n* stands for the number of objects B under consideration.

## 3. Results and Discussion

### 3.1. Mean-Squared Displacement

The main goal of our work was to determine changes in the ratio between the self-diffusion coefficient of a mixture of highly mobile molecules (solvent, designated as A) and those of reduced mobility (designated as B) as their concentrations in the solution change. In such systems, we can consider the short- and long-time self-diffusion coefficients for both types of particles A and B. This is related to the frequently asked question of how short-time diffusion, often associated with direct and hydrodynamic interactions of individual elements, influences the behavior observed in the long time. The self-diffusion coefficient, according to Einstein’s theory, can be related to the mean-squared displacement (MSD) of moving objects *Dr*(*t*)^2^ in following way:

(1) $$\langle \Delta r{\left(t\right)}^{2}\rangle =\frac{1}{n}\sum_{i=1}^{n}{\left[r_{i}\left(t\right)-r_{i}\left(0\right)\right]}^{2}=4Dt$$

where ***r****_i_*(*t*) denotes the space coordinates of the *i*th bead at time *t* and *n* is the number of movable elements.

**Figure 2:**
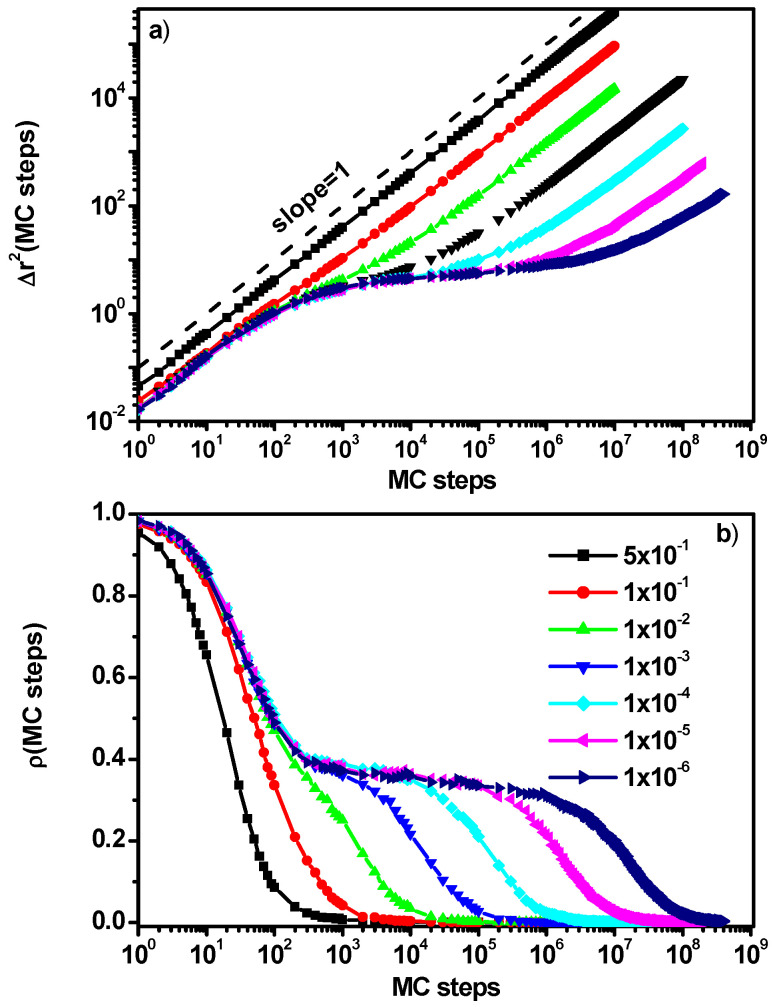
Mean-squared displacement of solvent (A) molecules as a function of the number of Monte Carlo steps (**a**) and the solvent position correlation function ρ(t) (**b**). The case for component B concentration of 0.5. Probabilities of motion of particles B are given in the inset.

Figure 2a shows examples of mean-squared solvent displacements obtained at a concentration of 0.5 of particles with reduced mobility (B) whose probability of participating in cooperative motion was systematically reduced. It can be observed that in the case under consideration, when the probability of B particles’ motion is not too limited, the solvent molecules exhibit almost Fickian diffusion in the entire range of the considered times, that is, MSD depends linearly on time [37]. Further reduction in the probability of motion of the B component leads to the appearance of a region of diffusion slowdown, the longer the lower the mobility of the B particles. Generally, in the case of MSD of solvent molecules (A), three regions can be distinguished: the first at short-time, in which diffusion is Fickian (I), the second in which we observe diffusion slowdown, the duration of which depends on the degree of slowdown of the B particles (II), and the last one, in which the solvent molecules return to Fickian diffusion (III). The diffusion process, where MSD does not depend linearly on time, but MSD ~ *t^a^*, where *a* < 1 is called subdiffusion, has been intensively studied in order to find its physical origin and determine its characteristics [50,53,54,55,56,57,58,59,60,61,62]. This behavior can be confirmed by the behavior of the position correlation function defined as:

(2) $$\rho \left(t\right)=\frac{1}{n}\sum_{i=1}^{n}m_{i}\left(0\right)m_{i}\left(t\right)$$

where *m_i_*(*t*) = 1 or 0, depending on if the ith object occupies or does not occupy its original position after time *t*, respectively. Therefore, this function gives the information on whether or not the ith object occupied its original position (at *t* = 0) and at a given time *t*, respectively. In Figure 2b, a close relationship can be observed between the relaxation behavior of the *r* function and the dynamical regimes observed in Figure 2a. In the case when the reduction in the probability of B particles is small, we observe a single relaxation process associated with a change in position. With the decrease in the probability of B particles’ motion, we observe an extension of the period of solvent particles’ (A) confinement by their neighbors corresponding to the diffusion slowdown region, and then a relaxation process associated with the return to normal diffusion. In this kind of system, the question always arises on whether the solvent molecules will exhibit normal diffusion in the long time. This stems from the fact that the B particles in the system under consideration constitute a kind of obstacle. We studied this problem in [51] and concluded that, regardless of the obstacle’s slowdown, the diffusion of A molecules remains normal in the long time. However, in the limiting case of stationary obstacles, subdiffusion behavior is observed after exceeding the percolation threshold [35].

The return of solvent molecules to normal diffusion is well illustrated by the behavior of the so-called non-Gaussian parameter *α*_2_(*t*) [57]. It shows how the distribution of object displacement deviates from the Gaussian distribution, and thus, it is a good measure of the dynamic heterogeneity of systems studied. The non-Gaussian parameter is defined as follows:

(3) $$\alpha_{2}\left(t\right)=\frac{\langle ∆r^{4}(t)\rangle }{2{\langle ∆r^{2}(t)\rangle }^{2}}-1$$

This parameter takes a value close to zero when the diffusion of the objects is close to Fickian diffusion, otherwise, the parameter value differs significantly from 0.

**Figure 3:**
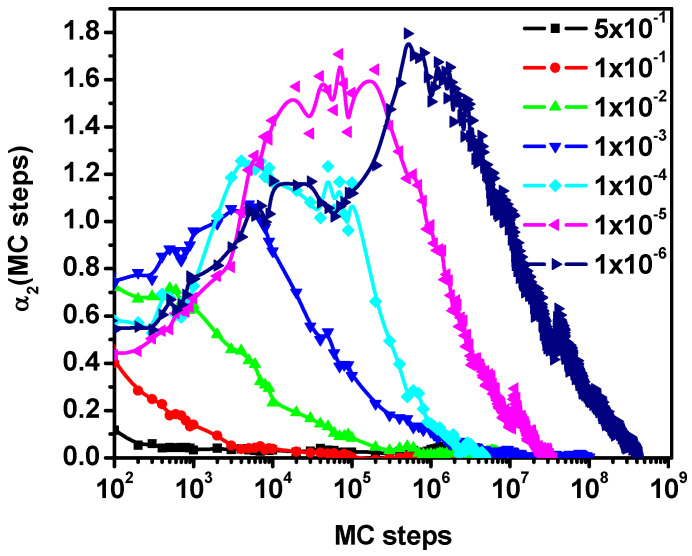
The non-Gaussian parameter *α*_2_(*t*) as a function of the number of Monte Carlo steps. The case for component B concentration of 0.5. Probabilities of motion of particles B are given in the inset.

The *a*_2_(*t*) parameter allows for estimating approximate changes in the nature of diffusion of A molecules observed in Figure 2a,b with the increase in the concentration of particles B. Figure 3 shows the non-Gaussian parameter as a function of time for solvent molecules when the concentration of B particles with reduced mobility is 0.5 (a variant corresponding to Figure 2) for various motion probabilities. It can be seen that in the regions corresponding to the diffusion slowdown, *α*_2_(*t*) increases rapidly and then decreases, tending to zero for long times, which indicates a return to Fickian diffusion. A more extensive discussion of this behavior of solvent molecules can be found in [52]. The results presented in Figure 3 and the results of [52] lead to a rather unambiguous conclusion that if particles B are treated as moving obstacles for molecules of type A, then regardless of how much we reduce the mobility of particles B, the diffusion of molecules A will return to normal diffusion after a sufficiently long time.

**Figure 4:**
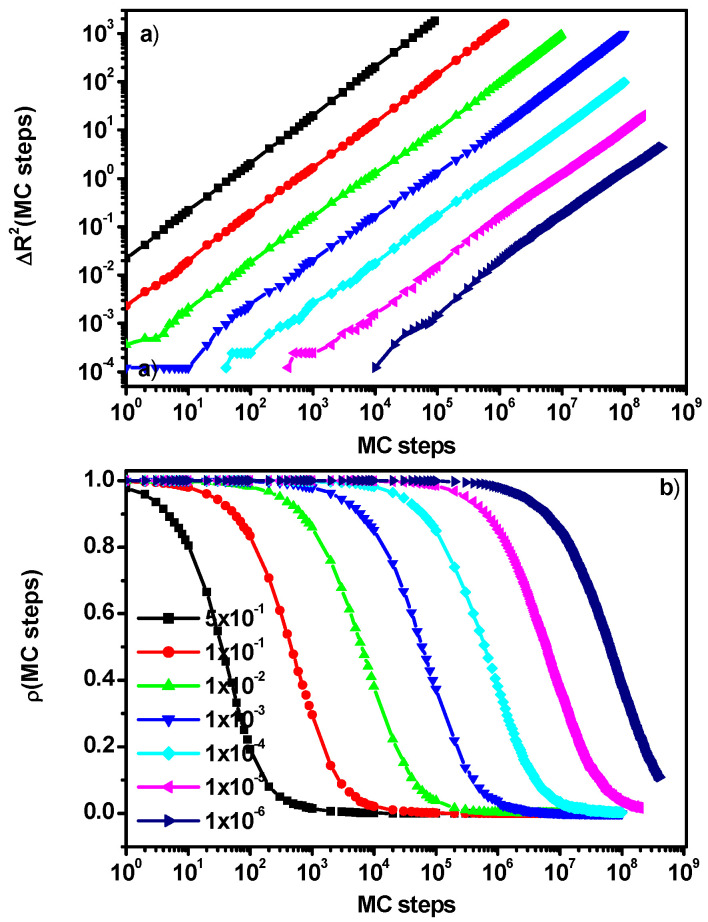
Mean-squares displacement of particles B as a function of the number of Monte Carlo steps (**a**) and the B particles’ position correlation function ρ(t) (**b**). The case for component B concentration of 0.5. Probabilities of motion of particles B are given in the inset.

Figure 4a shows an example of the mean-squared displacement of particles with reduced mobility (B) as a function of the number of Monte Carlo steps. Similarly, to the case of solvent molecules, the concentration of molecules with reduced mobility is 0.5, and the probability of participating in a cooperative motion loop varies between 0.5 and 10^−6^. In this case, unlike the situation considered for solvent molecules, we do not observe any special behavior that changes with time. In virtually all cases, it is impossible to distinguish regions in which the nature of diffusion would change. Particles B perform Brownian motion; their diffusion decreases with the change in the probability of participating in cooperative motions, but no effects of interaction with A molecules in the form of diffusion slowing nor other effects are observed. This behavior is confirmed by the position relaxation function shown in Figure 4b, where we observe a single, clearly visible relaxation process associated with a change in position. Over the entire time range, even in the case of a maximum reduction in the mobility of particles B, we do not observe trapping of these molecules by neighbors.

### 3.2. Diffusion Coefficient

It should be remembered that the diffusion coefficient can therefore be determined if the MSD dependence on time is linear. In the case of dense systems composed of objects being obstacles or with different mobility, this dependence takes the form *t^a^* where *a* < 0; we then have subdiffusion [62,63,64]. The short- and long-time self-diffusion coefficients are thus determined from the initial and long-time behavior of the MSD as

(4) $$D_{SH}=\underset{t\to 0}{\lim}\frac{\langle \Delta r^{2}\left(t\right)\rangle }{4t}\;\;\;\;\;and\;\;\;\;\;D_{LG}=\underset{t\to \infty }{\lim}\frac{\langle \Delta r^{2}\left(t\right)\rangle }{4t}$$

Using the MSD(*t*) relationships shown in Figure 2a and Figure 4a, the diffusion coefficients of both types of objects *D_A_* and *D_B_* can be determined using Equation (4). In this case, solvent diffusion coefficient can be determined for both regions where the diffusion is normal, i.e., for short time and long time (the subdiffusion phenomenon does not occur for solvent A, so in this case, we are dealing with only one diffusion coefficient value). Then, we calculate the reduced values of these coefficients *D_SH_A_*(*ϕ_B_*)*/D_A_*(0) and *D_LG_A_*(*ϕ_B_*)*/D_A_*(0), where *D_A_*(0) denotes the diffusion coefficient determined at zero concentration of B component, i.e., for pure solvent.

**Figure 5:**
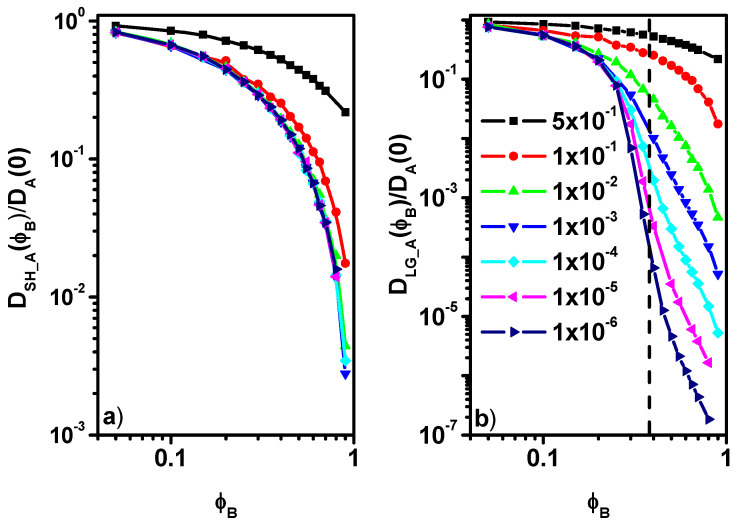
The ratio of short-time diffusion coefficient *D_SH_A_(ϕ_B_)* to diffusion coefficient at zero concentration of the B component *D_A_*(0) (**a**) and the ratio of long-time diffusion *D_LG_A_(ϕ_B_*) coefficient to the diffusion coefficient at zero concentration of B component *D_A_*(0) (**b**). The vertical dotted line indicates the location of the percolation point determined for the system with fixed obstacles (see text for details).

**Figure 6:**
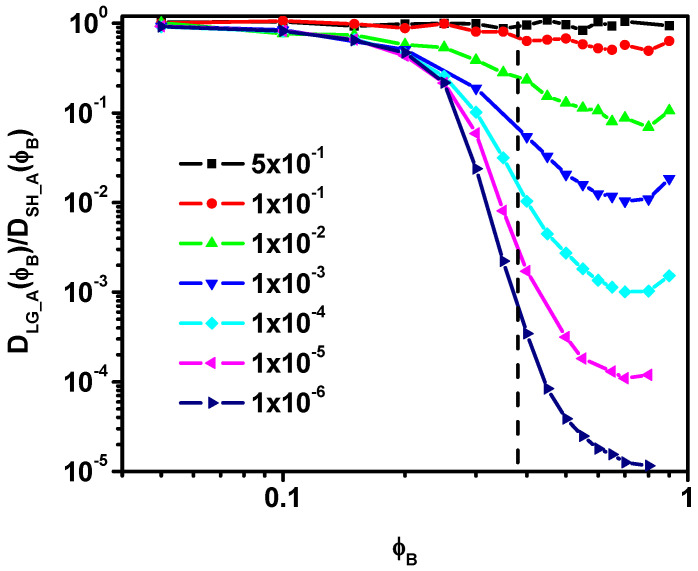
The ratio of long-time diffusion *D_LG_A_(ϕ_B_)* coefficient to the short-time diffusion coefficient *D_SH_A_(ϕ_B_)* as a function of *ϕ_B_*. The vertical dotted line indicates the location of the percolation point determined for the system with fixed obstacles.

Now, we will move on to discussing the behavior of the reduced diffusion coefficient of component A. Figure 5a shows the ratio of short-time diffusion coefficient *D_SH_A_* to the diffusion coefficient *D_A_*(0), corresponding to zero concentration of particles B with reduced mobility. It can be observed that for a small reduction in the B particles’ mobility (probability of motion 0.5 and 0.1), the curves describing *D_SH_A_(ϕ_B_)/D_A_*(0) as a function of the concentration of particles B differ from each other, and with a decrease in the probability of motion, this ratio decreases. However, for a greater reduction in the probabilities (0.01-10^6^), the curves describing the *D_SH_A_* *ϕ_B_)/D_A_(0)* ratio overlap, i.e., after exceeding the reduction threshold of 0.01, the local diffusion behavior of the A molecules does not change, the short-time diffusion is practically the same, and it is independent of the reduction in the probability of motion of the particles B. This behavior is consistent with the MSD results presented in Figure 2a where in the case of a large reduction in the probability of the B particles’ motion (≤ 10^−2^), the MSD in the short time is practically independent of how the B particles move. The results obtained in the case of short-time diffusion for A molecules indicate that in the case of a large decrease in the mobility of particles B, the ratio *D_SH_A_(ϕ_B_)/D_A_*(0) can be described by a certain universal function *f(ϕ_B_)*. The behavior of the ratio *D_LG_A_(ϕ_B_)/D_A_*(0) is different in the case of long time. Here, we observe that when the probability of the motion of particles B is reduced to a small extent, the diffusion coefficient of A molecules systematically decreases with increasing *ϕ_B_*. Further reduction in the mobility of particles B leads to more abrupt changes in the *D_LG_A_(ϕ_B_)/D_A_*(0) ratio. Changes in the reduced long-time solvent diffusion coefficient *D_LG_A_(ϕ_B_)/D_A_*(0) with the colloid concentration *φ_B_* are presented in Figure 5b. In this Figure, we have also marked the percolation threshold value determined for a similar model but with fixed obstacles, which was determined in [19] (for systems with an obstacle concentration above the percolation threshold, solvent diffusion does not return to normal and its movement is restricted). The behavior of *D_LG_A_(ϕ_B_)/D_A_*(0) is fundamentally different from *D_SH_A_(φ_B_)/D_A_*(0), which indicates that there is no simple relationship between these quantities. This fact is well illustrated in Figure 6, which shows the *D_LG_A_/D_SH_A_* ratio as a function of *ϕ_B_*. The decrease in solvent long-time diffusion coefficient with the concentration of colloid is considerably more rapid than for short-time and faster than that found in an experiment (optical microscopy) and Monte Carlo simulations of hard disks where the relationship is linear [17,65]. Non-monotonic behavior in the relative diffusion coefficient was observed for rods in networks when rods’ length approached the network mesh size; this behavior was attributed to lowered entropic free energy barrier [66,67]. However, in our model, there is no network in which moving objects would interact, although there are less mobile colloid particles of the same size that act as obstacles. There are no interactions in our model (except for the excluded volume), so it is difficult to determine the effect of solvent motion on the temporary “barriers” composed of colloid particles. In earlier studies, we examined the waiting time for movement, and it was evident that the nature of blocking or trapping moving objects is rather Arrhenius-like [33,46]. It was also shown that the excluded volume and HI are responsible for the large reduction in diffusion of larger objects [31].

It can be observed that as the mobility of the B component decreases, the shape of the ratio *D_LG_A_/D_SH_A_* presented in Figure 6 changes significantly. For example, with a small reduction in the probability of motion (0.5 and 0.1), the *D_LG_A_/D_SH_A_* ratio as a function of *ϕ_B_* remains approximately constant (this would probably require further studies and discussion). When the mobility of particles B is further reduced, the shape of *D_LG_A_/D_SH_A_* as a function of concentration becomes more complex. In this case, three regions can be distinguished. The region I corresponds to low *ϕ_B_* concentrations (0 ≤ *ϕ_B_* ≤ 0.2) where the *D_LG_A_/D_SH_A_* ratio remains approximately constant and has similar values regardless of the degree of reduction in the mobility of particles B. In the region II (0.2 < *ϕ_B_* ≤ 0.80), we observe a sharp decline in the ratio *D_LG_A_/D_SH_A_*, which becomes more rapid the greater the reduction in the mobility of particles B molecules. This behavior can be related to approaching and exceeding the percolation point in the considered model. The region III, 0.80 < *ϕ_B_*, in which we observe slight increases in the considered dependence, is related to the fact that at very high *ϕ_B_* concentrations, due to the close correlation of the motion of objects A and B, the mobility of A molecules decreases when compared to the mobility of B. Perhaps at very high colloid concentrations, the motion of both species becomes coupled through the formation of cooperative loops. Here, long-time diffusion of the mobile solvent may be severely limited by its dependence on slow collective rearrangement dominated by slow colloidal particles, while short-time solvent diffusion may still reflect slightly higher mobility. This would imply that the observed increase in the *D_LG_A_/D_SH_A_* ratio could result from their different scaling as the colloid concentration approaches 1.

**Figure 7:**
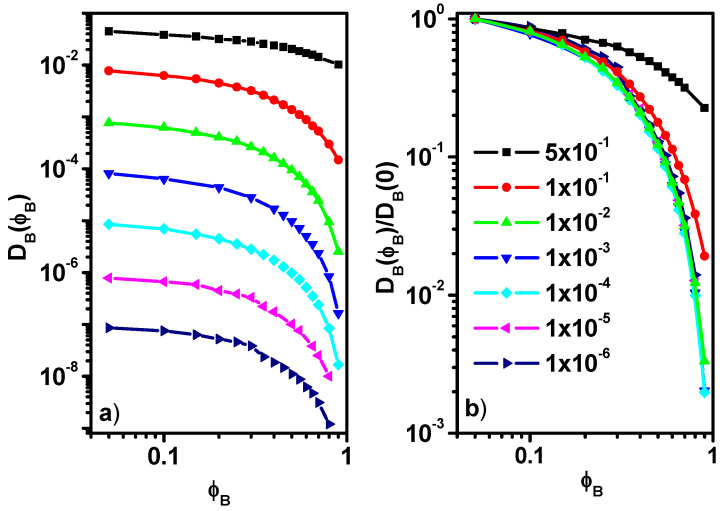
Absolute diffusion coefficients *D_B_(ϕ_B_)* as a function of *ϕ_B_* (**a**) and the ratio *D_B_(ϕ_B_)/D_B_(0)*, where *D_B_(0)* is the diffusion coefficient of particles B at infinite dilution (**b**).

Now we move on to discussing the diffusion coefficients of particles B. Figure 7a shows the absolute diffusion coefficients *D_B_(ϕ_B_)* as a function of *ϕ_B_*, while Figure 7b shows the relative values *D_B_(ϕ_B_)/D_B_(0)*, where *D_B_(0)* is the diffusion coefficient of particles B at infinite dilution. In the case of particles B, only diffusion coefficients for long times are presented because, according to the results presented in Figure 4, in this case, we do not observe any clear differences between diffusion at short and long times, manifesting, for example, in diffusion slowdown. The presented dependence of the *D_LG_A_/D_SH_* ratio of colloid concentration is therefore different from previous experimental and simulation findings, where no stabilization of this ratio occurs for low *φ_B_* values and the decrease in this ratio is almost linear [17].

In Figure 7a, two characteristic behaviors of the diffusion coefficients of particles B can be observed with the change in their concentration *ϕ_B_* in the system. At low concentrations below the percolation threshold, the decrease in the absolute value of the diffusion coefficients is not very rapid but becomes faster above the mentioned threshold. The observed differences between the individual curves are mainly caused by an arbitrary decrease in the probability of motion of particles B. Figure 7b presents the behavior of the *D_B_(ϕ_B_)/D_B_(0)* ratio as a function of the *ϕ_B_*. The behavior of this ratio shows similarity to that observed in the case of *D_SH_A_(ϕ_B_)/D_A_(0)*, e.g., in the case of a greater reduction in the mobility of particles B, we observe a situation in which *D_B_(ϕ_B_)/D_B_(0)* can be described by one universal function *g*(*ϕ_B_*). The dynamics of colloidal particles was also studied for systems containing athermal [42] and interacting models [65], where an exponential decrease in the diffusion coefficient with concentration was demonstrated.

**Figure 8:**
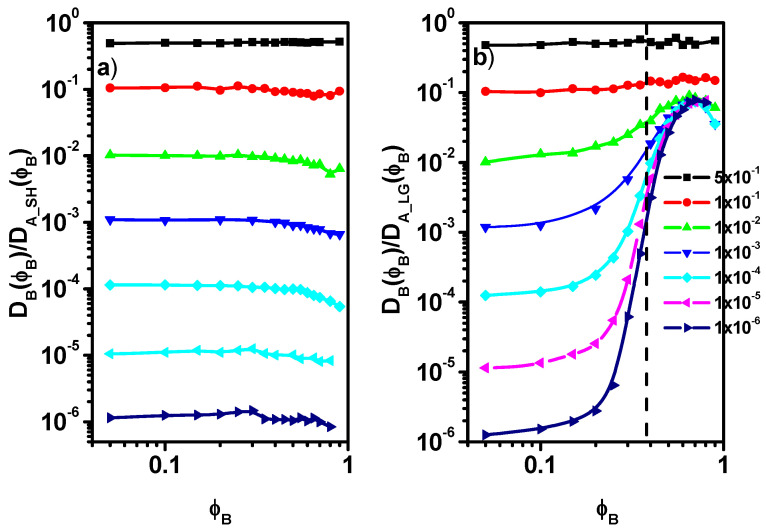
The ratio *D_B_(ϕ_B_)/D_A_SH_(ϕ_B_)* (**a**) and the ratio *D_B_(ϕ_B_)/D_A_LG_(ϕ_B_)* (**b**) as fuhnction of *ϕ_B_* for different values of the probability of motion of particles B. The vertical dotted line indicates the location of the percolation point determined for the system with fixed obstacles.

Some important conclusions regarding the dynamics of the considered system can also be drawn by examining the behavior of the solvent diffusion coefficients for short-time *D_SH_A_(ϕ_B_)* and long-time *D_LG_A_(ϕ_B_)* comparing with the case of the diffusion coefficient *D_B_(ϕ_B_)* of particles B. Figure 8a shows the *D_B_(ϕ_B_)/D_A_SH_(ϕ_B_)* ratio as a function of *ϕ_B_*. It can be seen that in all cases, this ratio remains practically constant over the entire range of concentrations *ϕ_B_* considered.

The observed differences result from an arbitrary reduction in the probability of particles B motion. This suggests a close relationship between the short-time diffusion of the solvent and the long-time diffusion of particles B. Figure 8b presents the *D_B_(ϕ_B_)/D_A_LG_(ϕ_B_)* ratio as a function of the concentration *ϕ_B_*. One can observe a slow increase in this ratio in the case of 0.05 < *ϕ_B_* < 0.30 which becomes more rapid as the percolation threshold is approached and exceeded, i.e., for 0.30 < *ϕ_B_* < 0.70. For B concentration *ϕ_B_* > 0.70 a decrease in this ratio is observed. The increase *D_B_(ϕ_B_)/D_A_LG_(ϕ_B_)* in the 0.05 < *ϕ_B_* < 0.70 region, first slow and then faster, is related to the fact that due to the close correlation of the motions of both types of molecules, the long-time diffusion of particles B increases relative to the long-time diffusion of A molecules, which is reduced in this process. At high concentrations of *ϕ_B_* > 0.70, the close correlation in the motion leads to a slight decrease in *D_B_(ϕ_B_)/D_A_LG_(ϕ_B_)*. Similar behavior, i.e., a constant ratio of the diffusion coefficient of the chain mass center to the short-time diffusion coefficient of the solvent [49], indicates a certain universality in these behaviors.

### 3.3. Motion in a Cooperative Loop

In the DLL model we used, the single source of both short- and long-time dynamics are cooperative motion loops. Without going into detail, they represent the result of both direct and hydrodynamic interactions. Therefore, analyzing the distribution of the loop of motion lengths and the probabilities of participation of the considered elements seems perfectly justified.

**Figure 9:**
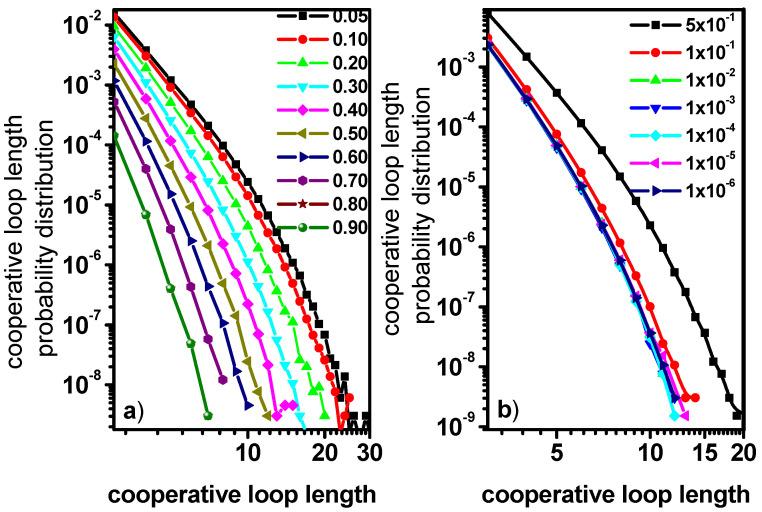
The cooperative loop length distribution for various concentration of B particles and for the probability of participating in a cooperative move reduced by 0.5 (**a**) and for various reductions in B particles’ mobility at the concentration of B particles *ϕ_B_* = 0.5 (**b**) as a function of the loop length.

Figure 9 shows the probability distribution of cooperative length loop obtained as a result of the process described in the Model and Methods section for A and B objects. In Figure 9a, the presented distributions were determined for different concentrations of particles B with the probability of their mobility reduced by 0.5. In Figure 9b, the distributions concern different mobilities of particles B at their concentration *ϕ_B_ =* 0.5. It has to be noted that the cooperative motion loops presented in Figure 1 arise exclusively as a result of excluded volume interactions; no other interactions were taken into account and such a procedure is of a completely basic nature. In the case of the results presented in Figure 9a, we can notice that, for low concentrations of particles B, the distribution of loop lengths is very wide and loops with lengths between 3 and 35, which indicates the long-range and many-body nature of the interactions. As the concentration of particles B increases, the probability distributions of the cooperative motion loop lengths change, decreasing the probability values and eliminating loops of greater length. This is the result of an increase in the concentration of molecules with limited mobility, which led to the screening of interactions. Figure 9b presents the results for the case where the concentration of particles B with reduced mobility is constant *ϕ_B_* = 0.5 but their mobility changes. Here, it can be noticed that when the probability of motion of the colloid particles is less than 0.10, all distribution curves converge. This behavior is completely different from that of solvent motion in a matrix of immobile obstacles, where the probability of participating in a loop of a given length decreases regularly with increasing obstacle concentration [35]. The behavior presented in Figure 9b seems surprising considering the results presented in Figure 2a for the solvent molecules, where the differences between the MSD(t) curves for individual values of the probability of motion are rather large. However, it should be noted that similar behavior was observed for the relative diffusion coefficients presented in Figure 5a (short-time diffusion of A molecules) and Figure 7b (long-time diffusion of particles B). Therefore, we can probably see the effect of introducing hydrodynamic interactions within the transport model introduced by the DLL algorithm in the behavior of short-time solvent diffusion and long-time colloid diffusion.

Another quantity that allows for a more general description of the statistics of cooperative movement loops is the total probability of bead motion *P_tot_* which could be defined as the total number of cooperative movements *N_tot_* performed by all beads in the entire system divided by the number of simulation steps *R* and by the size of the system (in a two-dimensional lattice system represented by *L^2^*):

(5) $$P_{tot}=\frac{N_{tot}}{RL^{2}}$$

**Figure 10:**
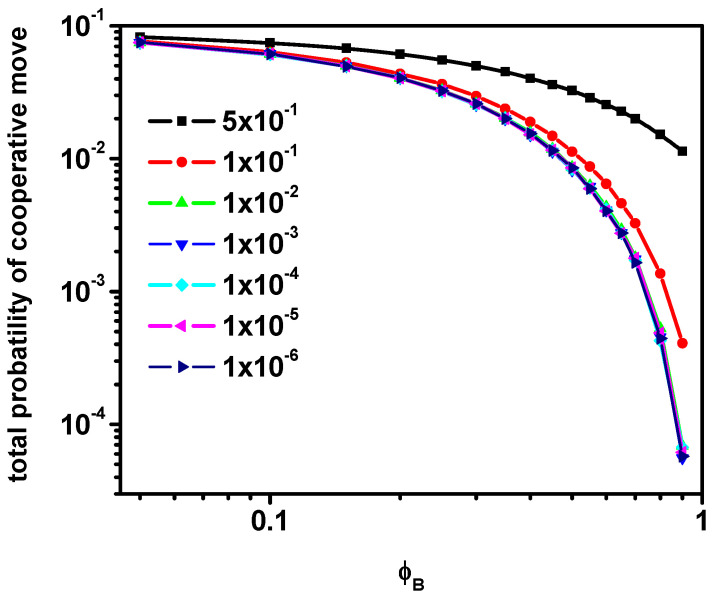
The total probability of the movement in cooperative move for various reduction in B particles’ mobility as a function of *ϕ_B_*. Probabilities of motion of B particles are given in the inset.

Figure 10 shows the total probability of the participation in the cooperative motion loop of objects in the system for different mobilities of particles B as a function of their concentration. It can be observed that, similarly to the case of short-time diffusion of A molecules and relative diffusion of particles B, the curves coincide when the reduction in the mobility of particles B is greater than 0.

## 4. Conclusions

In this work, we performed computer simulations of model colloidal systems to investigate their dynamics. Our main goal was to establish the relation between diffusion coefficients (in the short and long time) associated with the elements considered as solvent molecules and colloid particles. Strictly two-dimensional systems were approximated on a triangular lattice, and the solutions contained two types of objects: solvent molecules and colloidal particles. We assumed that both colloid particles and solvent molecules had the same size and shape, while we assumed that colloid particles had reduced mobility over a wide range. The systems studied were dense, i.e., completely filled with objects. The model was athermal, and the only potential was the excluded volume of the objects, while the hydrodynamic interactions in the studied systems arose from the assumption of cooperative movers of objects. These motions were implemented using the Dynamic Lattice Liquid algorithm, where objects change their position by participating in cooperative motion loops.

The study showed that the anomalous solvent diffusion (subdiffusion) that occurred was only temporary and that normal diffusion returned in all cases; the greater slowing down of colloidal particles only led to a longer period of subdiffusion. Differences in short- and long-time diffusion of both species were also demonstrated. In the case of a decrease in the mobility of colloid particles, two clearly visible relaxation processes related to the solvent diffusion can be observed, separated by a time interval in which the diffusion slows down. In this case, the nature of the relative diffusion coefficient (the short-time diffusion coefficient scaled by the diffusion coefficient of pure solvent) as a function of colloid particle concentration depends on the reduction in colloid particle mobility. In the case of a small reduction, a clear change in the relative diffusion coefficient is observed with the reduction (the curves are separated). In the case of further reduction in the colloid mobility, the curves describing relative diffusion coefficients overlap. This indicates that after reaching a certain threshold in solvent concentration, further reduction in the mobility of colloid particles has no effect on the movement of the solvent in the short time. The situation is different in the case of solvent diffusion over a long time, where the relative diffusion coefficient decreases with increasing concentration of colloid particles, but the reduction in their mobility clearly affects the nature of the curves. In the case of the ratio of the long-time and short-time diffusion coefficient, we observe a significant influence of the colloid particle mobility on its character. Moreover, this ratio does not decrease over the entire range of concentrations considered: for high concentrations of colloid particles, we observe an increase in the long-time diffusion coefficient compared to the short-time diffusion coefficient, which should be attributed to the close correlation in the motion of individual elements. In the case of colloid particles, the relative diffusion coefficient (the short-time diffusion coefficient scaled by the diffusion coefficient of pure solvent) behaves similarly to the analogous relationship for solvent molecules, i.e., there is a mobility threshold for colloid molecules for which the curves overlap. The reduction in relative diffusion coefficients (values relative to the diffusion of pure solvent) proved to be similar for short-time diffusion of solvent molecules and long-time diffusion of colloid particles. Similar behavior was already found for the diffusion of oligomers in the explicit solvent.

## Data Availability

The raw data supporting the conclusions of this article will be made available by the authors on request.
